# Layer-by-Layer Assembly and Electrochemical Study of Alizarin Red S-Based Thin Films

**DOI:** 10.3390/polym11010165

**Published:** 2019-01-18

**Authors:** Wei Ma, Yanpu Zhang, Fei Li, Donghui Kou, Jodie L. Lutkenhaus

**Affiliations:** 1 State Key Laboratory of Fine Chemicals, Dalian University of Technology, Dalian, Liaoning 116023, China; koudonghui1993@mail.dlut.edu.cn; 2 Artie McFerrin Department of Chemical Engineering, Texas A&M University, College Station, TX 77843, USA; yanpuzhang@tamu.edu (Y.Z.); fli2020@gmail.com (F.L.); 3 Department of Materials Science and Engineering, Texas A&M University, College Station, TX 77843, USA

**Keywords:** Alizarin Red S, layer-by-layer assembly, UV–Vis spectrometry, electrochemistry

## Abstract

Electroactive organic dyes incorporated in layer-by-layer (LbL) assemblies are of great interest for a variety of applications. In this paper, Alizarin Red S (ARS), an electroactive anthraquinone dye, is employed to construct LbL (BPEI/ARS)_n_ films with branched poly(ethylene imine) (BPEI) as the complementary polymer. Unconventional LbL methods, including co-adsorption of ARS and poly(4-styrene sulfonate) (PSS) with BPEI to assemble (BPEI/(ARS+PSS))_n_, as well as pre-complexation of ARS with BPEI and further assembly with PSS to fabricate ((BPEI+ARS)/PSS)_n_, are designed for investigation and comparison. Film growth patterns, UV–Vis spectra and surface morphology of the three types of LbL assemblies are measured and compared to reveal the formation mechanism of the LbL films. Electrochemical properties including cyclic voltammetry and spectroelectrochemistry of (BPEI/ARS)_120_, (BPEI/(ARS+PSS))_120_ and ((BPEI+ARS)/PSS)_120_ films are studied, and the results show a slight color change due to the redox reaction of ARS. ((BPEI+ARS)/PSS)_120_ shows the best stability among the three samples. It is concluded that the manner of dye- incorporation has a great effect on the electrochemical properties of the resultant films.

## 1. Introduction

Layer-by-layer (LbL) assembly is a simple and powerful method to fabricate multilayer films for a variety of applications [1,2,3,4,5]. Polycations and polyanions are usually used as assembly species [6,7,8,9], but other building blocks, including proteins [10,11,12,13], polysaccharides [14,15,16], nanoparticles [17,18,19], and dyes [20,21], have also been investigated. The inclusion of organic dyes into multilayer films is of great interest for functional films [22,23]. The challenge with many of these dyes is that they are small molecules, so their assembly into films is less energetically favored. Dubin explored similar challenges with surfactants and organic dyes, both in terms of partitioning and in assembly [24,25,26,27,28,29].

Electroactive dyes are responsive to electrical stimulation with a reversible variation of one or more physico-chemical properties [30]. They show wide technological applications in electrochemical and biological sensing, electrocatalysis, photovoltaic and electrochromic areas [31]. Immobilizing electroactive dyes using LbL assembly technique is an attractive way to incorporate the functionality and responsiveness of the dyes onto a surface of choice. Electroactive dyes, including phthalocyanine dyes [32,33,34,35,36], porphyrin dyes [37,38,39], methylene blue [40] and naphthol green B [41], have been layer-by-layer assembled with other species, and the obtained multilayer films were studied for potential use as electrochemical devices.

**Figure 1:**
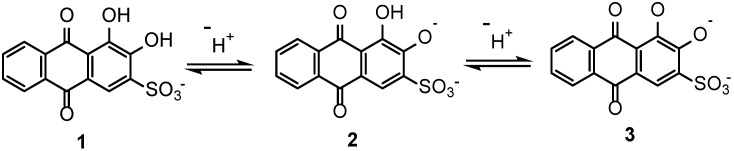
Chemical structure of Alizarin Red S (**1**) and its ionized structures (**2** and **3**).

Anthraquinone-based dyes are also of interest for their electroactivity. Due to their relevance in biological systems and technical use, the redox behavior of anthraquinones has been studied extensively [42,43,44,45]. Alizarin Red S (1,2-dihydroxy-9,10-anthraquinone-3-sulfonate, ARS, Figure 1), is one such type of electroactive anthraquinone dye. It is of particular interest for its electrochromic properties, along with its strong color contrast [46]. Due to the presence of electron donor (phenolic) and electron acceptor (quinone) moieties, ARS is electrochemically amphoteric, which is especially interesting from the point of view of molecular electronics [47]. It has been used as an electrochemical sensor, electrochemical indicator, and reducing agent [48,49,50,51,52,53,54]. ARS bears a negative charge, so it can be expected to assemble with polycations to form new types of electrochemically functional nanostructures. Its incorporation in multilayer films may show different properties for various applications, such as electrochemical sensors and electrochromic films. However, very few studies of assembly of ARS LbL deposited films and their resulting electrochemical properties have been reported probably due to the fact that ARS is a singly charged small molecule.

ARS contains several functional groups: besides sulfonic groups, ARS has hydroxyl and carbonyl groups. The hydroxyl groups ionize to provide certain electrostatic interaction sites, and hydrogen bonding can form between amino-containing polycations and ARS. In addition, the large planar structure of the anthraquinone may also facilitate anchoring on the surface to promote LbL construction.

**Scheme 1:**
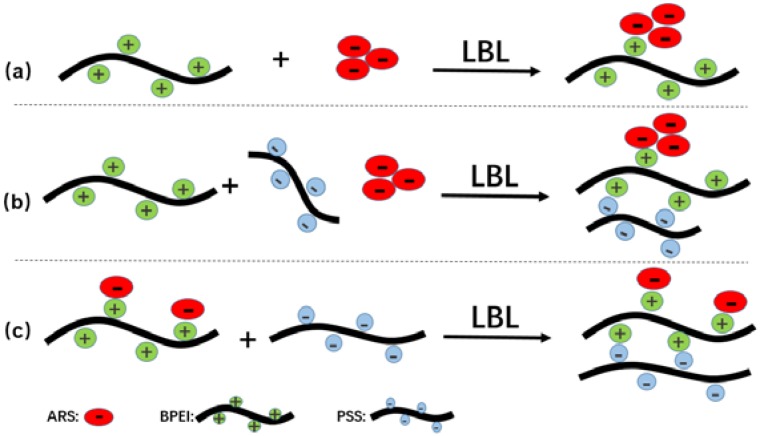
Schematics of LbL assemblies in this study: (**a**) (BPEI/ARS)_n_, (**b**) (BPEI/(PSS+ARS))_n_ and (**c**) ((BPEI+ARS)/PSS)_n_.

In this paper, branched polyethyleneimine (BPEI) was used as the complementary polycation for LbL assembly, and the properties of (BPEI/ARS)_n_ LbL films on ITO-coated glass were studied. Besides the conventional LbL assembly method, which commonly refers to the fabrication of building blocks into multilayer thin films without prior or post treatment [55], unconventional LbL methods with more than one step in assembly, are also important for incorporation of more compositions and for producing more functionalities [56,57]. In this study, ARS was also incorporated in the multilayers through two unconventional LbL methods. One was the co-adsorption of ARS and poly(4-styrenesulfonate) (PSS) onto BPEI to obtain (BPEI/(PSS+ARS))_n_ films in which ARS and PSS competitively adsorb onto the BPEI layer; the other was pre-complexation of ARS with BPEI, and then the pre-complex was alternately adsorbed with PSS to obtain ((BPEI+ARS)/PSS)_n_ films (Scheme 1).

The key idea of these unconventional approaches is that they include more than one step in the assembly process. It is anticipated that such LbL methods will provide useful routes to supramolecular architectures with more structural complexity for more robust usage. The film growth, UV–Vis spectrum, surface morphology and electrochemical properties of the three kinds of films were studied and compared. The knowledge gained from these studies will provide useful information for future strategies to develop functional hybrid materials based on dye-incorporated LbL assemblies.

## 2. Experimental Section

### 2.1. Chemicals and Materials

Branched poly(ethylene imine) (BPEI, *M*_w_ = 25,000) and Alizarin Red S (ARS) were purchased from Sigma Aldrich (St. Louis, MO, USA). Polystyrene sulfonate (PSS, *M*_w_ = 500,000) was obtained from Scientific Polymer Products, INC. (Ontario, NY, USA). Other chemicals were of analytical grade or higher and used without further purification. Aqueous solutions were prepared using 18.2 MΩ cm (Milli-Q) water. Indium-tin oxide (ITO) coated glass was purchased from Delta Technologies (Loveland, CO, USA).

### 2.2. Preparation of LbL Assemblies

(BPEI/ARS)_n_, ((BPEI+ARS)/PSS)_n_ and (BPEI/(PSS+ARS))_n_ LbL assemblies were prepared on ITO-coated glass slides using a programmable slide stainer (HMS series, Carl Zeiss Inc., Thornwood, NY, USA). ITO-coated glass slides were cleaned by immersion in a H_2_O-NH_4_OH-H_2_O_2_ (5:1:1) mixture at 70 °C for 15 min followed by sonication-cleaning in Milli-Q water three times for 5 min each. After cleaning, the ITO-coated glasses were dried using high-velocity nitrogen gas. Plasma-cleaning (Harrick PDC-32G) was then carried out for 5 min.

For (BPEI/ARS)_n_ film, the ITO-coated glass slides were immersed in pH 5.0 BPEI solution for 15 min followed by three rinses with Milli-Q water for 2, 1 and 1 min each. Then, the substrates were immersed in pH 5.0 ARS solution for 15 min, followed by another three water rinses for 2, 1 and 1 min each. The cycles were repeated n times to form a (BPEI/ARS)_n_ LbL film. For ((BPEI+ARS)/PSS)_n_ film, the ITO-coated glass slides were first immersed in pH 5.0 solution containing both BPEI and ARS for 15 min, the rinse processes were the same as that used for (BPEI/ARS)_n_ assembly. Then, the substrates were immersed in pH 5.0 PSS solution for another 15 min, followed by the same rinse procedures. The cycles were repeated n times to form a ((BPEI+ARS)/PSS)_n_ LbL film. For (BPEI/(PSS+ARS))_n_ film, the procedures were almost the same as that described above, except that ARS was mixed with PSS, not BPEI, and the solutions of BPEI and (ARS+PSS) were both kept at pH 5.0. The concentrations of both polymers and ARS were 1 mg·mL^−1^, and 0.5 M of NaCl was used for rinse baths. The LbL films were dried in a convection oven at 70 °C for 10 min and stored in a sealed vial until further use.

### 2.3. Characterization

The thickness of the LbL films was measured using profilometry (P-6, KLA-Tencor, Milpitas, CA, USA). The films were scored to the substrate surface using a razor blade and step heights were measured at five different locations and averaged. Surface morphology and roughness were characterized in the dry state via atomic force microscopy (AFM) using a Dimension icon System Nano IR2-s (Bruker Nano. Inc., Billerica, MA, USA) in tapping mode. HQ: NSC35/Al BS AFM probes (MikroMasch, Watsonville, CA, USA) with a typical tip radius of 8 nm were used. UV–Vis spectroscopy was performed using a SolidSpec-3700 UV-VIS-NIR spectrometer. The electrochemical tests were carried out using a three-electrode system inside a 1.00 cm cuvette. Silver wire with 0.5 mm diameter (Alfa Aesar, Haverhill, MA, USA) was used as the quasi-reference electrode (QRE), and platinum wire of 0.3 mm diameter (Alfa Aesar) as the counter electrode. LbL films on ITO-coated glass were used as working electrodes (Delta Technologies, Loveland, CO, USA). Cyclic voltammetry was performed at room temperature using Gamry Instrument (Interface 1000, Warminster, PA, USA) in the voltage range of −1.0–0.2 V versus Ag QRE. The spectroelectrochemical measurements were obtained using a combination of the Gamry and SolidSpec-3700 spectrometer, and also employing the above mentioned three-electrode electrochemical system inside a 1.00 cm cuvette. The film on the non-conducting sides of the ITO-coated glasses was wiped away, and the conducting side with the film was centered in the cuvette to assure equal light intensity for both front and backside illumination. The supporting electrolyte was an aqueous solution of 0.1 M NaCl. The electrochemical cell was held at 25 °C. The Ag QRE was calibrated with respect to the potassium ferricyanide/potassium ferrocyanide (Fe(CN)_6_^3−^/Fe(CN)_6_^4−^) couple. The half-wave potential (E_1/2_) of Fe(CN)_6_^4−^/Fe(CN)_6_^3−^ measured in 0.1 M NaCl solution was 0.18 V vs. Ag QRE. Fe(CN)_6_^4−^/Fe(CN)_6_^3−^ = 0.36 V vs. SHE (standard hydrogen electrode). Thus, the potential of the Ag wire was assumed to be 0.18 V vs. SHE.

## 3. Results and Discussion

### 3.1. Digital Images and UV–Vis Spectroscopy

**Figure 2:**
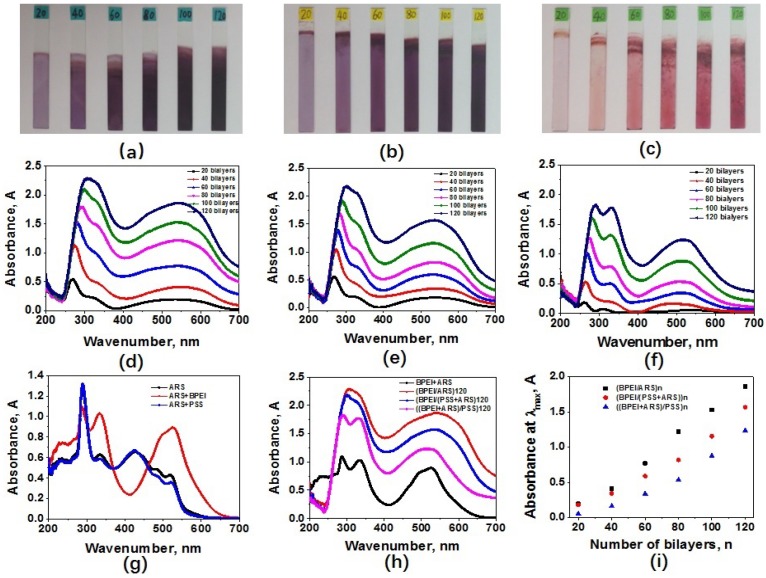
Photographs of (**a**) (BPEI/ARS)_n_, (**b**) (BPEI/(ARS+PSS))_n_ and (**c**) ((BPEI+ARS)/PSS)_n_ on ITO-coated glass; UV–Vis spectra of (**d**) (BPEI/ARS)_n_, (**e**) (BPEI/(ARS+PSS))_n_ and (**f**) ((BPEI+ARS)/PSS)_n_ on ITO-coated glass; (**g**) UV–Vis spectra of ARS, ARS+BPEI and ARS+PSS; (**h**) UV–Vis spectra of BPEI+ARS solution, (BPEI/ARS)_120_, (BPEI/(ARS+PSS))_120_ and ((BPEI+ARS)/PSS)_120_ on ITO-coated glass; (**i**) Relationship of the number of bilayers or layers pairs and the absorbance of the films at the maximum absorption wavenumber.

The digital photographs of (BPEI/ARS)_n_, (BPEI/(ARS+PSS))_n_, and ((BPEI+ARS)/PSS)_n_ for when n was 20, 40, 60, 80, 100 and 120 are presented in Figure 2a–c, respectively. For all three kinds of assemblies, the colors of the films gradually became deeper, indicating that more dye was incorporated as the number of layer pairs increased. BPEI showed a positive charge because of partial protonation of the amino groups at pH 5. As ARS and PSS contained sulfonic groups, the molecules exhibited negative charges. Dissociation constants for the two phenolic hydroxyl groups in ARS were reported as pK_1_(2-OH) = 5.49 and pK_2_(1-OH) = 10.85 [46]. At pH 5, about 20% of 2-OH of ARS ionized to form 2-O^−^ [46], so ARS also contains phenolic anion groups. Mainly due to the electrostatic interactions, (BPEI/ARS)_n_ and (BPEI/(ARS+PSS))_n_ films grew and more dye was introduced with the increase of the layer number. ((BPEI+ARS)/PSS)_n_ also grew, indicating the positive charge of the complex (BPEI+ARS).

**Figure 3:**
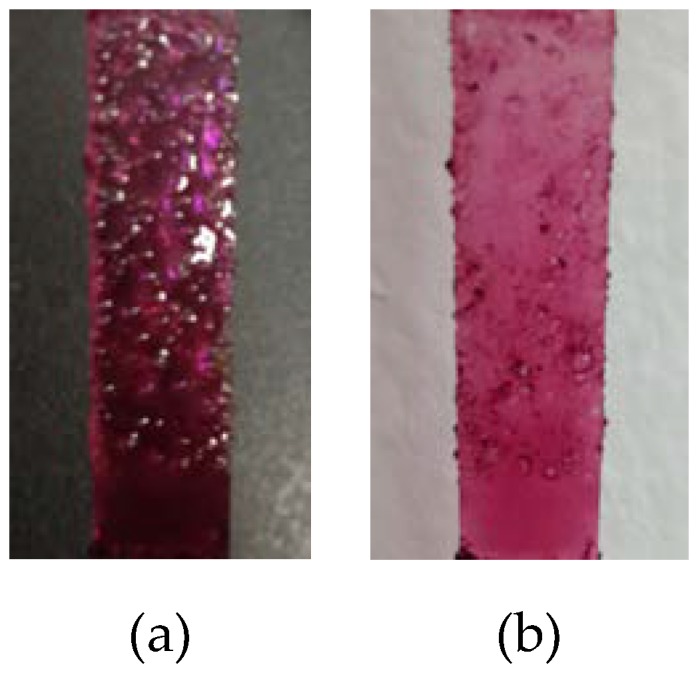
Hydrogel formed during assembly of ((BPEI+ARS)/PSS)_120_ under wet conditions, (**a**) dark background and (**b**) bright background.

However, the color shade and color strength differed among the three assembly methods. ARS solution was orange yellow at pH 5, while (BPEI/ARS)_n_ and (BPEI/(ARS+PSS))_n_ films were dark purple, and ((BPEI+ARS)/PSS)_n_ was peach red. The film colors were far from that of the ARS solution, which is mainly owing to the bathochromic effect of ARS’s interaction with BPEI. In addition, it can be observed that the (BPEI/ARS)_n_ and (BPEI/(ARS+PSS))_n_ films appeared much smoother even when the number of layer pairs was large; however, ((BPEI+ARS)/PSS)_n_ films appeared rougher and rougher as the number of layer pairs increased. A special phenomenon of hydrogel formation was observed only during the assembly of ((BPEI+ARS)/PSS)_n_ films (see Figure 3). When ARS was added to BPEI solution, physical crosslinking among BPEI molecules formed with ARS, which contains multifunctional groups. Then in LbL assembly, the crosslinked (BPEI+ARS) molecules adsorbed on the film, and then interacted with the adsorbed PSS to expand the spatial network, resulting in formation of the red hydrogel; after drying, the hydrogel collapsed into a film with a rough surface.

The color differences among the different assemblies were also monitored using UV–Vis spectroscopy (Figure 2d–f). The maximum absorption wavelengths (λ_max_) of (BPEI/ARS)_n_ and (BPEI/(ARS+PSS))_n_ films within the visible region were quite close to each other, located at about 540 nm (Figure 2d,e), while that of ((BPEI+ARS)/PSS)_n_ was at about 520 nm (Figure 2f). Such λ_max_ values indicated that the films were all red in color and that the color of (BPEI/ARS)_n_ and (BPEI/(ARS+PSS))_n_ films was deeper than that of ((BPEI+ARS)/PSS)_n_. Besides λ_max_, the half peak width in the visible spectra also determines the color. It could be seen from Figure 2d–f that (BPEI/ARS)_n_ and (BPEI/(ARS+PSS))_n_ films showed broad peaks, while ((BPEI+ARS)/PSS)_n_ showed narrower one, indicating that the colors of the former were much darker and that of the latter was much brighter. These results were in accordance with the color of the films we observed.

The UV–Vis spectra of ARS, ARS+PSS and ARS+BPEI solutions were also measured for comparison (Figure 2g), they gave λ_max_ at 425, 425 and 525 nm, respectively. Both ARS and ARS+PSS showed 3 absorption peaks at 425, 480 and 520 nm in the visible region, which were assigned to the absorption of ARS and ARS with partially ionized hydroxyl groups (Figure 1). As ARS and PSS are both anionic molecules and their intermolecular interaction is small, addition of PSS in ARS solution shows little influence on the spectra and the color. However, when BPEI was added to the ARS solution, the visible spectrum showed a bathochromic shift and a notable hyperchromic effect, indicating strong interaction between the molecules. Dyes with significant planarity usually associate with each other to form a packed structure. Due to the electrostatic interaction and hydrogen bonding between ARS and BPEI, the π–π stacking among the dye molecules was hindered and a *J*-type aggregate between ARS and BPEI formed [58].

By comparing the UV–Vis spectra of the solutions, distinct differences can be seen from that of the assembled films. When ARS or ARS+PSS was assembled with BPEI on ITO-coated glass, visible absorption spectra of the assemblies showed an obvious bathochromic shift compared with that of ARS solution, indicating intermolecular interaction between the ionic species, resulting in a *J*-type aggregate. Spectroscopy methods have confirmed the formation of dye molecular aggregates in LbL films. The formed aggregates can be significantly different from those commonly observed in aqueous solution [58,59,60].

The changes in the UV spectra were not as obvious as that in the visible spectra- ARS displays three obvious bands at 252, 288 and 325 nm (Figure 2g), which are *p*-p* transitions with benzenoid, quinonoid and benzenoid characters, respectively [61,62,63]. Complexation of ARS and BPEI increases absorption at about 330 nm due to a dye-disassociation effect. For (BPEI/ARS)_n_, (BPEI/(ARS+PSS))_n_, and ((BPEI+ARS)/PSS)_n_ assemblies (Figure 2d–f), the peaks at around 280 and 330 nm still exist, and the peaks at about 280 nm show an obvious red shift with increasing layer pair number, indicating the interaction of anthraquinone with BPEI.

In the co-adsorption process, ARS and PSS competed with each other to interact on BPEI; complexation of PSS with BPEI reduced the number of amine interaction sites for ARS, thus leading to less dye adsorbed. However when ARS was precomplexed with BPEI and then assembled with PSS, the spectra of the ((BPEI+ARS)/PSS)_n_ films showed a similar shape and close λ_max_ compared with that of (ARS+BPEI) solution (as presented in Figure 2h). It can be estimated that only some of the BPEI was precomplexed with ARS as BPEI contains many more cationic groups. This is the reason why much less dye was adsorbed for ((BPEI+ARS)/PSS)_n_ assemblies. As PSS did not show much interaction with the dye, the visible spectra of ((BPEI+ARS)/PSS)_n_ was much the same as that of (ARS+BPEI) solution. These results also revealed that the color of ((BPEI+ARS)/PSS)_n_ could be predicted according to that of (BPEI+ARS) solution.

From Figure 2h, it can clearly be seen that (BPEI/ARS)_120_ films showed the largest dye incorporation amount, (BPEI/(ARS+PSS))_120_ much less, and ((BPEI+ARS)/PSS)_120_ the least. For other layer pairs, the comparison results are the same (see Figure 2i). In all cases, the absorbance increased almost in proportion to the increase of the number of the layer pairs, indicating regular growth of the dye layers.

### 3.2. Film Growth Behavior

**Figure 4:**
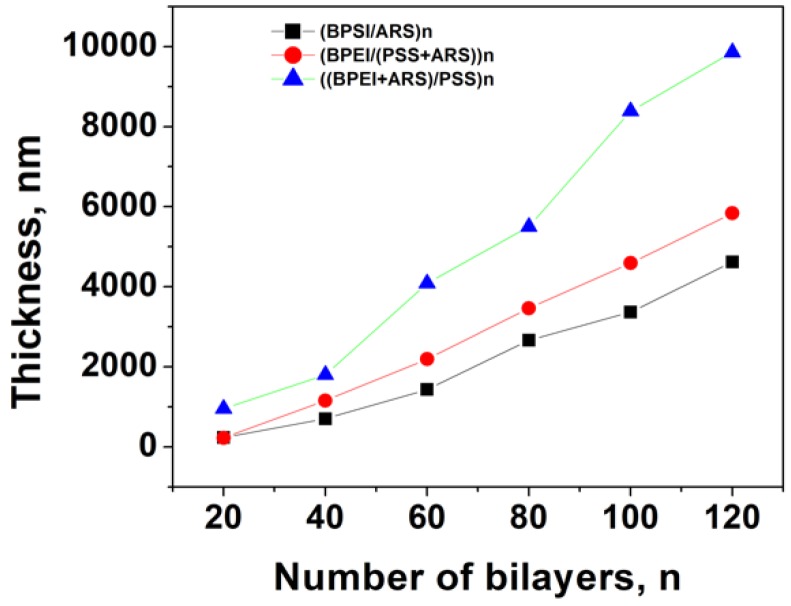
Relationship between the number of bilayers (or layer pairs) and thickness for when n is 20, 40, 60, 80, 100 and 120.

The thicknesses of the (BPEI/ARS)_n_, (BPEI/(PSS+ARS))_n_ and ((BPEI+ARS)/PSS)_n_ films were measured using a profilometer for when n was 20, 40, 60, 80, 100 and 120 (Figure 4). All three types of assemblies showed linear growth behavior, indicating suppressed interlayer diffusion throughout the multilayer films [64]. When the bilayer number was 20, the thicknesses of (BPEI/ARS)_20_, (BPEI/(PSS+ARS))_20_ and ((BPEI+ARS)/PSS)_20_ were 237, 226 and 955 nm, respectively; when the bilayer number increased to 120, the thicknesses of (BPEI/ARS)_120_, (BPEI/(PSS+ARS))_120_ and ((BPEI+ARS)/PSS)_120_ reached 4620, 5840 and 9860 nm, respectively. The thickness of ((BPEI+ARS)/PSS)_120_ was even double of that of (BPEI/ARS)_120_. The average layer pair thicknesses for (BPEI/ARS)_120_, (ARS/(PSS+ARS))_120_ and ((BPEI+ARS)/PSS)_120_ films were 38, 49 and 82 nm, respectively. The regular growth is attributed to the strong interactions among assembly species.

It can be seen that ((BPEI+ARS)/PSS)_n_ films showed the largest growth rate, (BPEI/(PSS+ARS))_n_ grew more slowly, and (BPEI/ARS)_n_ grew the slowest. These results were just opposite to the color depth we observed and the visible absorbance growth law at λ_max_ in Figure 2i, in which (BPEI/ARS)_n_ displayed the highest color strength and the largest absorbance increase rate, (BPEI/(PSS+ARS))_n_ showed the moderate rate values and ((BPEI+ARS)/PSS)_n_ the slowest ones. When assembling ((BPEI+ARS)/PSS)_n_ films, BPEI chains with the fewest number of associated ARS molecules likely adsorb preferentially, so the film grew very quickly. On the other hand for co-adsorption, when PSS was incorporated in the LbL films, the films grew quicker owing to that PSS was a polymer with a large amount of anionic groups and more interactions existed between PSS and BPEI vs. ARS (does it mean compared with ARS, PSS is more easy to adsorb on BPEI?). Although the growth rate of (BPEI/ASR)_n_ assembly was the lowest, its gradual increase to 4630 nm at 120 bilayers also revealed a significant molecular interaction between BPEI and ARS. Besides electrostatic attraction, hydrogen bonding and van der Waals force between BPEI and ARS were also participating.

### 3.3. Surface Morphology

**Figure 5:**
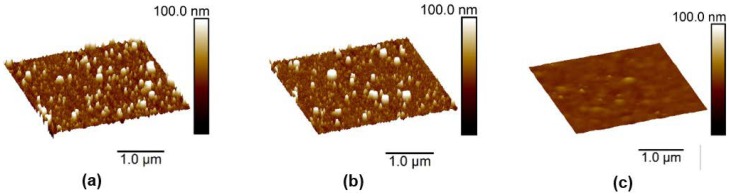
Atomic force microscopy 3D height images of (**a**) (BPEI/ARS)_20_, (**b**) (ARS/(PSS+ARS))_20_ and (**c**) ((BPEI+ARS)/PSS)_20_ films.

The surface morphology and roughness of the (BPEI/ARS)_20_, (ARS/(PSS+ARS))_20_ and ((BPEI+ARS)/PSS)_20_ films in the dry state were investigated using AFM (Figure 5). The root-mean-square (rms) roughness of each film surface, as calculated from AFM height images, was 17.2, 15.1 and 2.41 nm, respectively. Accordingly, we observed larger features on the film surface for the assembly with more dye incorporated when measuring the 20-layer pair films, namely, (BPEI/ARS)_20_ film surface showed the largest roughness, (ARS/(PSS+ARS))_20_ was less rough, while ((BPEI+ARS)/PSS)_20_ surface was quite smooth. For (BPEI/ARS)_20_ and (ARS/(PSS+ARS))_20_ surfaces, many small island-like features were observed. It is likely that the features are regions of clustered dye particles; similar particle clustering was observed in other layer-by-layer systems [35,65]. For ((BPEI+ARS)/PSS)_20_, as it contains quite a small amount of dye molecules, having mainly BPEI and PSS, the surface was much smoother. However, as discussed in the above section, with the increase of the number of layer pairs, we can clearly observe a rough surface of the ((BPEI+ARS)/PSS)_n_ due to collapse of the hydrogel.

### 3.4. Electrochemical Properties

The electrochemical behavior of (BPEI/ARS)_120_, (BPEI/(ARS+PSS))_120_, and ((BPEI+ARS)/PSS)_120_ films was investigated using cyclic voltammetry and spectroelectrochemistry.

#### 3.4.1. Cyclic Voltammetry

**Figure 6:**
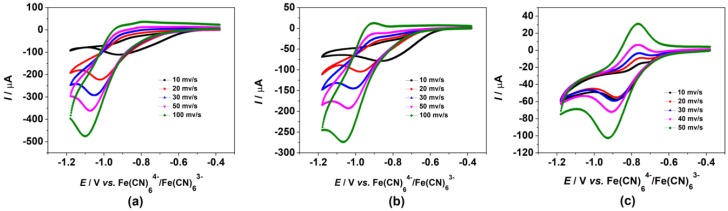
CVs of (BPEI/ARS)_120_ (**a**), (BPEI/(ARS+PSS))_120_ (**b**) and ((BPEI+ARS)/PSS)_120_ (**c**) on ITO-coated glass in 0.1 M NaCl.

**Figure 7:**
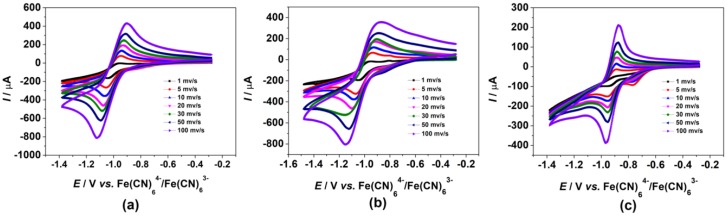
CVs of ARS (**a**), ARS+PSS (**b**) and ARS+BPEI (**c**) solutions in 0.1 M NaCl.

Cyclic voltammetry (CV) is a sensitive method for the characterization of anthraquinoids with regard to their electrochemical properties. In electrochemistry, anthraquinones exhibit a reversible 2-electron transfer process for its quinone/hydroquinone redox couple [66]. The two-electron reduction, depending on the availability of protons in the reaction media, can be accompanied by the uptake of two protons. In aqueous media, the reduction process is thus highly pH-dependent. As Milli-Q water was used, one hydroxyl group was present in dissociated form (Dye 2 in Figure 1), the reduction is likely accompanied by the uptake of one proton. (BPEI/ARS)_120_, (BPEI/(ARS+PSS))_120_, and ((BPEI+ARS)/PSS)_120_ films were subjected to CV over the potential range −0.38 to −1.18 V (vs. Fe(CN)_6_^4−^/Fe(CN)_6_^3−^) with different scan rates as shown in Figure 6. For comparison, CVs of ARS, ARS+PSS and ARS+BPEI solutions were also measured in Figure 7. The film-coated ITO glass or ITO glass was used as the working electrode (WE) vs. silver wire as the quasi-reference electrode (QRE) in 0.1 M NaCl against a platinum wire counter electrode (CE).

From the CVs in Figure 6, (BPEI/ARS)_120_ and (BPEI/(ARS+PSS))_120_ films showed an irreversible redox reaction as the anodic peaks were not obvious or very small as compared to the cathodic peaks, while ((BPEI+ARS)/PSS)_120_ showed a quasi-reversible redox reaction. The irreversible reaction is mainly due to the low stability of the (BPEI/ARS)_120_ and (BPEI/(ARS+PSS))_120_ films during cyclic scanning. Owing to the consumption of protons in the reactions consecutive to the cathodic electron transfer, the pH in the cathodic diffusion layer increases. As the films are assembled mainly through electrostatic interaction, they are sensitive to pH and unstable as pH increases. This leads to gradual film dissolution during CV measurements, resulting in the irreversible redox reaction. However, the redox reaction in the ((BPEI+ARS)/PSS)_120_ film was fairly stable, which was attributed to the thick film construction and the small amount of dye relative to the large amount of polymer. More protection from the polymers resulted in a reduced dissolution for the dyes.

In Figure 7, the solutions of ARS, ARS+PSS and ARS+BPEI all presented quasi-reversible redox reactions. Figure 7a shows a cathodic peak potential of *E*_pc_ = −1.0 to −1.1 mV for ARS solution, which is just in accordance with the cathodic peak potential observed for (BPEI/ARS)_120_ in Figure 6a. Another cathodic peak at −0.75 mV only appears at the low scan rate of 1 mV/s in Figure 7a. Both cathodic peaks for ARS solution were close to that reported in literature [46]. According to the literature, the cathodic peaks in Figure 6a are attributed to the reduction of ionized dye 2 and fully ionized dye 3 (as shown in Figure 1) which was produced due to the increase of pH in the boundary layer. The electrochemical reaction of (BPEI/(ARS+PSS))_120_ films was quite the same for that of (BPEI/ARS)_120_, except that (BPEI/(ARS+PSS))_120_ showed a more distinct anodic peak at −0.9 mV at higher scan rates of 50 and 100 mV/s, which was almost at the same position as that of *E*_pa_ in Figure 7a,b. This was also consistent with the anodic peak observed in literature for ARS solution at pH 5.02 [46]. For ARS+BPEI solution, the positions of *E*_pc_ and *E*_pa_ in the voltammogram were different from that for ARS solution. ARS+BPEI solution showed a cathodic peak of *E*_pc_ = −0.91 to −0.96 mV and an anodic peak of E_pa_ = −0.89 to −0.85 mV in Figure 7c. Clearly, the reduction potential of ARS+BPEI is higher than that of ARS and ARS+PSS, which was mainly attributed to the decrease in the electron density of anthranquinone resulting from precomplexation. The redox-peaks became much sharper, suggesting faster electron transport through the dye layer. For ((BPEI+ARS)/PSS)_120_, *E*_pc_ = −0.88 to −0.92 mV, which was also higher than that for (BPEI/ARS)_120_ and (BPEI/(ARS+PSS))_120_ due to precomplexation.

From the above investigation, it can be seen that due to the pH increase during the reduction, (BPEI/ARS)_120_ and (BPEI/(ARS+PSS))_120_ became unstable and resulted in an irreversible redox reaction. ((BPEI+ARS)/PSS)_120_ was quite stable during cyclic scanning, and electrochemical reduction became easier due to precomplexation.

#### 3.4.2. Spectroelectrochemistry

**Figure 8:**
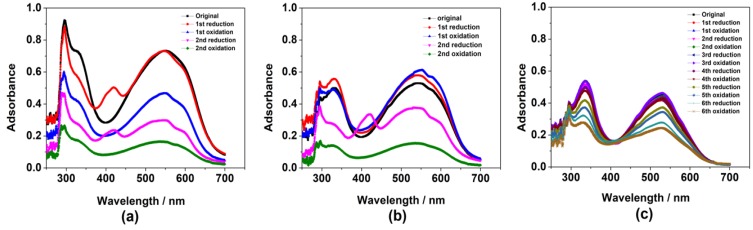
UV–Vis spectra of (**a**) (BPEI/ARS)_120_, (**b**) (BPEI/(ARS+PSS))_120_ and (**c**) ((BPEI+ARS)/PSS)_120_ LbL films in 0.1 M NaCl aqueous solutions: after the 1st and 2nd electrochemical reduction (at voltage of −1.18 V vs. Fe(CN)_6_^4−^/Fe(CN)_6_^3−^) and oxidation (at voltage of −0.38 V vs. Fe(CN)6^4−^/Fe(CN)_6_^3−^) processes.

The electrochromic properties of (BPEI/ARS)_120_, (BPEI/(ARS+PSS))_120_, and ((BPEI+ARS)/PSS)_120_ LbL films were evaluated using spectroelectrochemistry, a method based on in situ collection of UV–Vis absorption spectra for electrodes held at a certain potential within a cuvette. Beginning at −0.38 V, the LbL films were in their original state (as shown in Figure 8, original absorption lines), presenting broad absorbance in the visible range for (BPEI/ARS)_120_ and (BPEI/(ARS+PSS))_120_, and much narrower absorbance for ((BPEI+ARS)/PSS)_120_. A linear voltage sweep began and the electrode potential became more cathodic; then, the in situ UV–Vis spectra were measured at −1.18 V (vs. Fe(CN)_6_^4−^/Fe(CN)_6_^3−^), representing the 1st reduction absorption curve (as shown in Figure 8). After measurement, a reverse linear anodic sweep began and then the spectra was recorded at −0.38 V (vs. Fe(CN)_6_^4−^/Fe(CN)_6_^3−^), representing the 1st oxidation absorption curve (as shown in Figure 8). The 2nd, 3rd and other reduction and oxidation absorption peaks in Figure 8 were obtained by repeating the above procedures.

It can be seen from Figure 8a that a new peak appeared at 425 nm, which was consistent with the λ_max_ of ARS, revealing the dissociation of ARS from the BPEI as the color of the films turned light. Also, a small shoulder at about 600 nm was observed, which was presumably due to the reduction product of ARS^2−^ (dye 3 in Figure 1). In addition, by holding at fully reduced and oxidized states of the dye, the absorption peak (BPEI/ARS)_120_ decreased dramatically after the 1st oxidation. Due to production of OH^−^ during the reduction process, the film gradually dissolved, so when measuring the change in the 1st oxidation process, the absorbance of the film decreased greatly. In the 2nd reduction and oxidation peak measurements, the absorbance continually decreased. Film dissolution was caused by decreased electrostatic interactions. The case for the spectral change of the (BPEI/(ARS+PSS))_120_ film was similar to that of (BPEI/ARS)_120_. The difference was that after 1st reduction and oxidation process, the film did not show a dramatic decrease in absorbance, revealing better dye stability in the film. For ((BPEI+ARS)/PSS)_120_ film, even at fully reduced and oxidized states, the color was still quite stable even after 4-cycles of the redox reaction. In the 5th reduction and oxidation cycle, the UV–Vis absorbance began to decrease, indicating a loss of ARS dye from the film. Much higher retention of the dye is mainly due to its protection brought about by precomplexation. However, no obvious new absorption peak was observed due to precomplexation.

Based on the above spectroelectrochemistry investigation, we conclude that the color change of (BPEI/ARS)_120_ and (BPEI/(ARS+PSS))_120_ films happened due to an electrochemically induced redox reaction from the results of the visible spectrum change, but the color contrast was not obvious. This is because the original dark color brings some difficulty in recognizing slight color changes. In addition, electrochemically mediated dissolution of the LbL films containing ARS and polymers occurred at an applied cathodic potential, which affects the reversibility of the color change.

## 4. Conclusions

Electroactive Alizarin Red S has been successfully incorporated into ultrathin films to construct (BPEI/ARS)_n_, (BPEI/(ARS+PSS))_n_ and ((BPEI+ARS)/PSS)_n_ on ITO-coated glass using conventional and unconventional LBL methods. When BPEI was added to the ARS solution, the visible spectrum showed a bathochromic shift and a notable hyperchromic effect, indicating strong interactions between the molecules. The colors of the assembled films are quite different from that of the ARS solution. (BPEI/ARS)_n_ and (BPEI/(ARS+PSS))_n_ films were dark purple and ((BPEI+ARS)/PSS)_n_ ones were peach red. The different color of ((BPEI+ARS)/PSS)_n_ is mainly due to disassociation of the dye by BPEI in solution. All three types of assemblies showed linear film growth behavior, and ((BPEI+ARS)/PSS))_n_ films have the largest layer pair thickness, followed by (BPEI/(ARS+PSS))_n_, and then (BPEI/ARS)_n_. However, the color growth rate of the three assemblies is just opposite, indicating that (BPEI/ARS)_n_ LbL assemblies are beneficial for dye incorporation to the film as compared to the other two assembly methods. Electrochemical properties of (BPEI/ARS)_120_, (BPEI/(ARS+PSS))_120_ and ((BPEI+ARS)/PSS)_120_ films were also different. The results showed that color change happened due to cathodic reduction; however, different extents of color change occurred due to varying states of dye aggregation. In addition, dye dissolution occured during the reduction process, which also affected the electrochromic properties of the films. The most stable film, in terms of electrochemical performance was ((BPEI+ARS)/PSS)_n_.
